# Fatal Tuberculous Meningitis With Hydrocephalus and SIADH (Syndrome of Inappropriate Antidiuretic Hormone Secretion) in an Immunocompetent Adult With Miliary Tuberculosis: Diagnostic Challenges Associated With Language Barriers

**DOI:** 10.7759/cureus.111487

**Published:** 2026-06-25

**Authors:** Priyanta Banerjee

**Affiliations:** 1 General Internal Medicine, Hull University Teaching Hospital, Scunthorpe General Hospital, Hull, GBR

**Keywords:** delayed diagnosis, disseminated tuberculosis, hydrocephalus, hyponatremia, immunocompetent adult, language barrier, miliary tuberculosis, siadh, tuberculous meningitis

## Abstract

Tuberculous meningitis is a severe manifestation of disseminated tuberculosis associated with high morbidity and mortality. I report the case of a 30-year-old previously healthy immunocompetent immigrant who initially presented with fever, malaise, and rhinorrhoea. A significant language barrier limited history-taking, contributing to diagnostic delay. His condition rapidly progressed to acute hydrocephalus and syndrome of inappropriate antidiuretic hormone secretion (SIADH) in the context of concomitant miliary pulmonary tuberculosis. Despite prompt initiation of anti-tubercular therapy, high-dose corticosteroids, and neurosurgical intervention, his neurological status deteriorated, and he succumbed within a short period. This case highlights the importance of early recognition of tuberculous meningitis and its potentially life-threatening complications, particularly among patients from tuberculosis-endemic regions where the disease remains prevalent. It also underscores how communication barriers in healthcare may delay diagnosis and worsen outcomes, especially in countries like the United Kingdom, where tuberculosis is relatively uncommon.

## Introduction

Tuberculous meningitis is one of the most severe forms of extrapulmonary tuberculosis and remains a major cause of neurological morbidity and mortality. Although rare in the United Kingdom, delayed recognition is common because the presentation is variable and often nonspecific. The diagnostic challenge is compounded by the fact that tuberculous meningitis frequently coexists with systemic tuberculosis, most often pulmonary disease [1,2].

Hydrocephalus is a well-recognised complication that occurs in up to 80% of cases and often requires neurosurgical intervention [3-5]. Despite treatment, outcomes are frequently poor. Hyponatraemia is another important complication and is usually mediated by the syndrome of inappropriate antidiuretic hormone secretion (SIADH). Both hydrocephalus and SIADH contribute substantially to adverse outcomes when not recognised early [6,7].

## Case presentation

A 30-year-old previously healthy man from East Timor presented to the Accident and Emergency (A&E) department of a district general hospital with an unclear duration of fever, generalised weakness, and reduced oral intake. He had very limited English, spoke Tetun, and had only a basic knowledge of Portuguese, which significantly hindered history-taking. Multiple attempts were made to use language-line interpretation services, but Tetun was unavailable. Eventually, limited information was obtained through Portuguese interpretation.

He had migrated to the United Kingdom one year earlier, was not registered with a general practitioner, and reported no significant past medical history or regular medication use. On Day 1 of hospital admission, he described a five-day history of intermittent fever, rhinorrhoea, reduced appetite, and generalised weakness. Owing to a significant language barrier, he was unable to provide a more detailed history despite multiple attempts to obtain interpreter support.

The initial examination was unremarkable. His temperature was 37.8 °C, blood pressure 110/70 mmHg, and oxygen saturation 97% on room air. He was provisionally diagnosed with a viral illness. Initial laboratory investigations are summarised in Table 1.

**Table 1:** Initial laboratory investigations COVID-19 PCR: COVID-19 Polymerase Chain Reaction; RSV: Respiratory Syncytial Virus

Test	Result	Reference Range
White cell count	9.3 × 10⁹/L	4.0–11.0 × 10⁹/L
Neutrophils	8.33 × 10⁹/L	2.0–7.5 × 10⁹/L
Lymphocytes	0.43 × 10⁹/L	1.0–4.0 × 10⁹/L
Platelets	244 × 10⁹/L	150–400 × 10⁹/L
C-reactive protein	14 mg/L	<5 mg/L
Urea	3.1 mmol/L	2.5–7.8 mmol/L
Creatinine	85 µmol/L	64–104 µmol/L
Sodium	122 mmol/L	135–145 mmol/L
Potassium	3.8 mmol/L	3.5–5.0 mmol/L
Chloride	84 mmol/L	98–107 mmol/L
Serum glucose	7.0 mmol/L	3.9–7.8 mmol/L
COVID-19 PCR	Negative	Negative
Influenza A/B	Negative	Negative
RSV	Negative	Negative

The patient was managed with intravenous fluids, paracetamol, and the Sepsis Six pathway. Although discharge was initially planned on Day 1, he remained in the hospital due to social concerns.

During Days 2 and 3 of admission, his clinical condition gradually deteriorated. Assessment was challenging due to persistent communication difficulties, which limited accurate evaluation of evolving symptoms.

**Figure 1:**
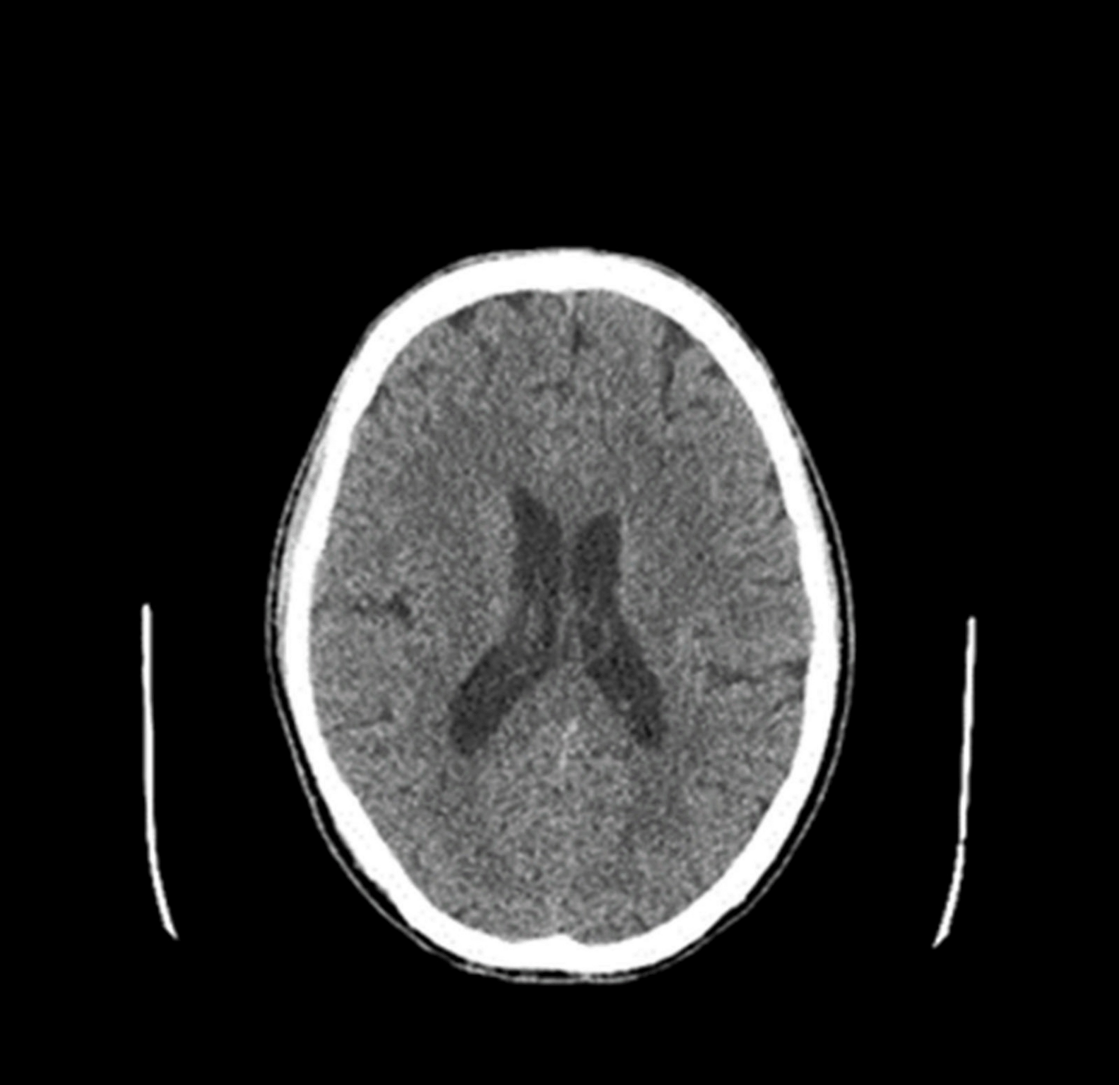
CT scan of the head prior to lumbar puncture showed no hydrocephalus or contraindications to lumbar puncture

On Day 4, he became drowsy, confused, and irritable, with a Glasgow Coma Scale (GCS) score of 12/15. Examination revealed marked photophobia, neck stiffness, and a positive Kernig's sign. A clinical diagnosis of meningitis was made, and empirical intravenous ceftriaxone and aciclovir were commenced. Computed tomography of the head, performed prior to lumbar puncture, showed no hydrocephalus or other contraindications to lumbar puncture (Figure 1). An urgent lumbar puncture was subsequently performed on the same day, and the results are summarised in Table 2.

**Table 2:** Cerebrospinal fluid findings consistent with meningitis CSF Analysis

Parameter	Result	Reference Range
Opening pressure	28 cmH₂O	6–20 cmH₂O
Appearance	Clear and colourless	Clear and colourless
White blood cells	100 cells/µL	0–5 cells/µL
Polymorphs	10 cells/µL	0 cells/µL
Lymphocytes	90 cells/µL	0–5 cells/µL
Gram stain	Negative	Negative
CSF PCR	Negative for common CNS viruses	Negative
CSF glucose	2.0 mmol/L	2.2–3.9 mmol/L
CSF protein	376 mg/dL	15–45 mg/dL

**Figure 2:**
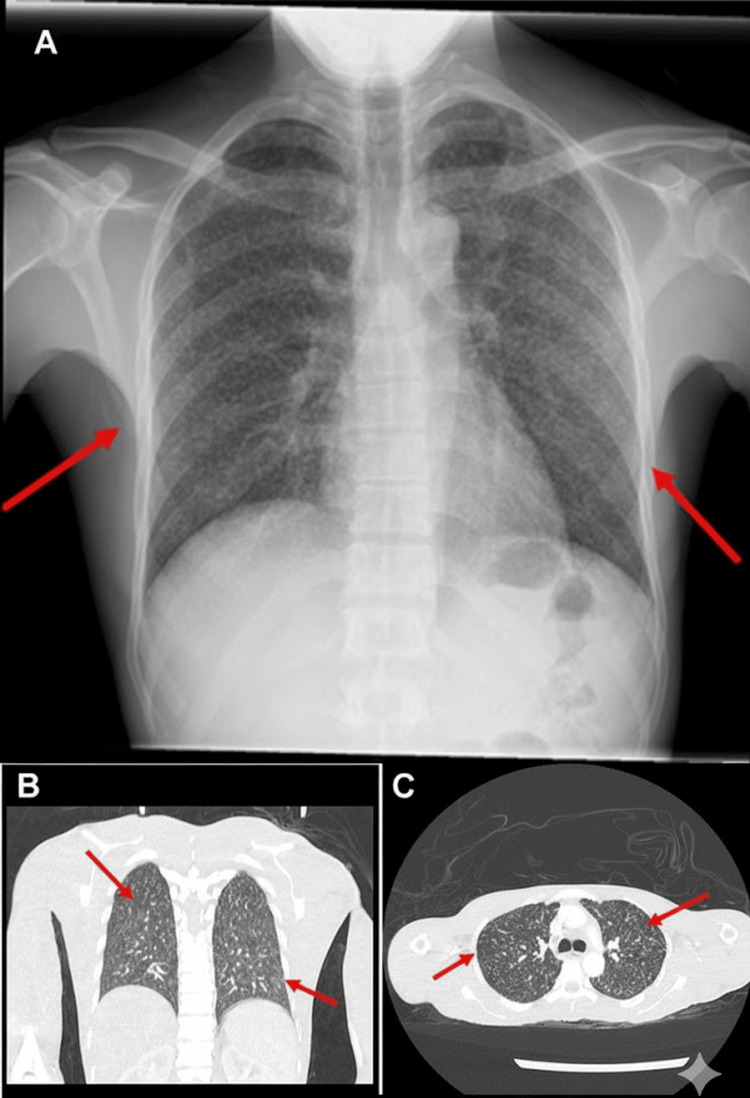
(A) Chest radiograph demonstrating diffuse bilateral miliary nodules. (B) Coronal CT chest showing numerous randomly distributed micronodules throughout both lungs. (C) Axial CT chest confirming a miliary pattern consistent with disseminated tuberculosis

Based on his CSF results, he was suspected to have tuberculous meningitis. Suspicion for tuberculous meningitis became more clinically plausible as the patient appeared cachectic, weighing 43 kg. Given his uncertain travel history, limited vaccination background, and clinical presentation, tuberculosis was considered likely. Chest radiographs were consistent with miliary tuberculosis, as shown in Figure 2.

Urgent microscopic examination of the CSF revealed acid-fast bacilli on Ziehl-Neelsen staining. Sputum microscopy also demonstrated AFB, and sputum PCR detected DNA fragments of *Mycobacterium tuberculosis*. He was started on isoniazid, rifampicin, pyrazinamide, and ethambutol, along with high-dose dexamethasone and proton pump inhibitor cover on Day 4. Subsequent CSF cultures confirmed growth of *Mycobacterium tuberculosis*, which was sensitive to conventional antitubercular therapy.

Hyponatraemia was present on admission (serum sodium 122 mmol/L) and persisted despite intravenous 0.9% sodium chloride administration during the first four days of admission. By Day 4, the serum sodium concentration had increased only marginally to 124 mmol/L, and the patient remained clinically euvolemic. SIADH secondary to tuberculous meningitis was therefore suspected, and subsequent biochemical investigations supported this diagnosis. Fluid restriction was initiated. Laboratory findings consistent with SIADH are summarised in Table 3.

**Table 3:** Laboratory findings consistent with SIADH TSH: Thyroid-Stimulating Hormone; SIADH: Syndrome of Inappropriate Antidiuretic Hormone Secretion

Parameter	Result	Reference Range
Serum sodium	124 mmol/L	135–145 mmol/L
Paired serum osmolality	270 mOsm/kg	275–295 mOsm/kg
Urine osmolality	566 mOsm/kg	50–1200 mOsm/kg
Urine sodium	77 mmol/L	<20 mmol/L
TSH	Normal	0.4–4.0 mIU/L
Cortisol	Normal	140–690 nmol/L

We also explored other potential sites of tuberculosis, but there was no evidence of disease elsewhere. It was unusual to see disseminated tuberculosis in someone who appeared immunocompetent, so an underlying immunocompromised state was considered. Basic immune screening, including HIV, hepatitis B and C, HbA1c, vitamin D, albumin, and an autoimmune panel, was unremarkable.

**Figure 3:**
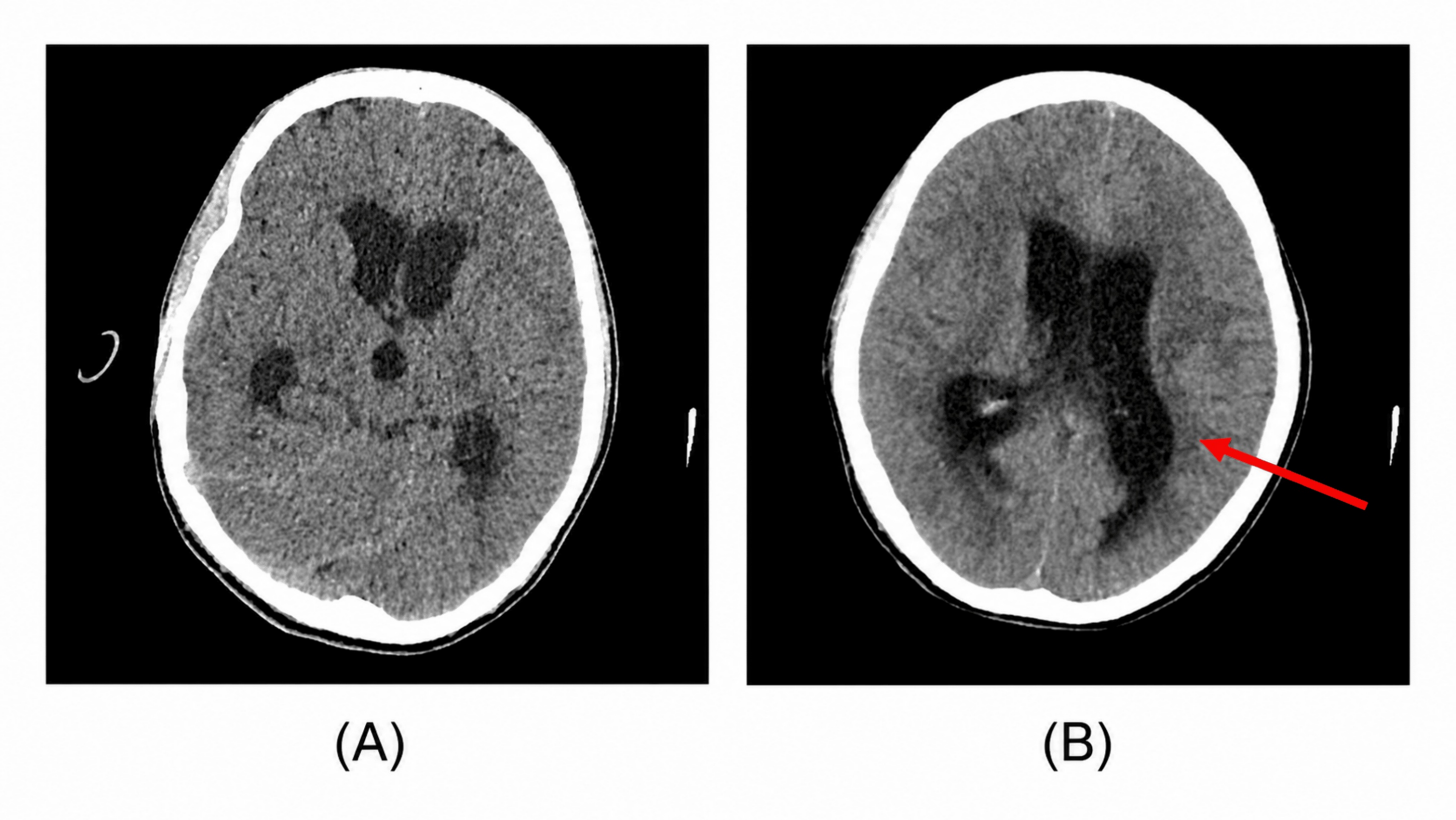
Repeat axial non-contrast CT head images demonstrating new-onset hydrocephalus. (A) Image at the level of the third ventricle showing dilatation of the lateral and third ventricles. (B) Higher axial image showing marked dilatation of the lateral ventricles. No definite focal obstructing lesion was identified on the selected images

Public Health England was informed, and on Day 5 of hospital admission, the patient was transferred from the district general hospital to a tertiary infectious diseases centre for further management. His GCS had been gradually declining, and it was 8/15 (E2, V2, M4) on arrival at the infectious diseases centre. On Day 6 of the hospital stay, an urgent repeat CT scan of the head was performed and demonstrated new-onset hydrocephalus (Figure 3).

No definite obstructive cause was identified, and the hydrocephalus was thought to be secondary to tuberculous meningitis. This represented an acute change compared with the CT head performed on Day 4 prior to lumbar puncture (Figure 1). This radiological deterioration developed within 48 hours. No additional neuroimaging had been performed in the intervening period.

Following the diagnosis of acute hydrocephalus on Day 6 of hospital admission, an urgent external ventricular drain (EVD) was inserted. He was subsequently transferred to the intensive care unit on the same day and required mechanical ventilation. Despite neurosurgical intervention, anti-tubercular therapy, corticosteroid treatment, and ongoing intensive care support, his neurological status failed to improve. He remained in the intensive care unit for five days, during which no meaningful neurological recovery was observed. Unfortunately, he subsequently died, and the case was referred to the coroner. As per his medical record, an autopsy was not done, and the rationale for not doing an autopsy was not explained in the patient's electronic record.

## Discussion

This case highlights the diagnostic difficulty of tuberculous meningitis in a low-incidence setting, particularly when the patient is apparently immunocompetent, and the initial history is limited by a language barrier. Although the patient had no recognised immunosuppression, he developed disseminated tuberculosis with miliary pulmonary involvement, microbiologically confirmed tuberculous meningitis, hydrocephalus, hyponatraemia consistent with SIADH, and a fatal neurological outcome. The case reinforces that severe disseminated tuberculosis should not be excluded solely because a patient is young or appears immunocompetent. Miliary tuberculosis results from haematogenous dissemination of *Mycobacterium tuberculosis*, and central nervous system involvement may occur through rupture or progression of a Rich focus [1,2].

The patient’s origin from Timor-Leste was clinically important. Timor-Leste remains a high-burden tuberculosis country, with the WHO reporting an estimated incidence of 498 cases per 100,000 population and 6,171 notified cases in 2023 [8]. This contrasts with England, where the UK Health Security Agency (UKHSA) reported 5,490 tuberculosis notifications in 2024 and an incidence of 9.4 per 100,000 population [9]. This epidemiological difference matters because clinicians in low-incidence settings may be less likely to suspect tuberculosis early, especially when symptoms are nonspecific, and communication is limited.

The main diagnostic clues in this case were not any single feature but the combination of epidemiological risk, cachexia, lymphopenia, marked hyponatraemia, evolving neurological signs, and supportive cerebrospinal fluid findings. Tuberculous meningitis often progresses subacutely, and one adult cohort reported a median symptom duration of three weeks before admission and a median time to diagnosis of five days after admission [10]. In contrast, this patient had a recognised symptom duration of approximately five days before admission, neurological deterioration within the first 24 hours of admission, and hydrocephalus within two days of an initially normal CT head. Treatment delay is clinically important, as delayed initiation of treatment after admission has been associated with increased mortality in tuberculous meningitis [11].

Hydrocephalus and SIADH were important complications. Hydrocephalus in tuberculous meningitis is usually caused by basal meningeal inflammation impairing cerebrospinal fluid flow or absorption, and it may develop despite appropriate antimicrobial therapy and corticosteroids [5]. A normal initial CT head should therefore not prevent repeat neuroimaging if consciousness declines. Hyponatraemia is also clinically important because it may worsen confusion, cerebral oedema, seizure risk, and neurological assessment. In this case, SIADH was favoured over cerebral salt wasting because the patient was clinically euvolaemic, with low serum osmolality, inappropriately concentrated urine, elevated urine sodium, and normal thyroid and adrenal function [6].

The language barrier was a central patient-safety issue. Tetum/Tetun interpretation was unavailable despite attempts to access language-line services, and communication was attempted through Portuguese, which was not the patient’s primary language. This limited the assessment of symptom duration, tuberculosis exposure, vaccination background, constitutional symptoms, and early neurological complaints. Migrant patients with tuberculosis may experience diagnostic delay related to healthcare access, communication barriers, and unfamiliarity with the health system [12]. More broadly, limited English proficiency can adversely affect healthcare access, use, and outcomes among immigrants [13]. Professional interpretation should therefore be viewed as a clinical safety intervention rather than an administrative task [14].

However, the language barrier should not be presented as the sole cause of the fatal outcome. The delay was likely multifactorial, involving nonspecific early symptoms, incomplete history, low background tuberculosis prevalence in the UK, reduced ability to report deterioration, ward pressures, reduced observation, lack of on-site infectious diseases and neurosurgical services, and the aggressive natural history of tuberculous meningitis. A single case report cannot quantify exactly how many hours would have been saved by correcting one factor. However, earlier integration of epidemiological risk, severe hyponatraemia, low body weight, and incomplete history could reasonably have accelerated senior review, chest imaging, tuberculosis testing, and infectious diseases input within the first 24 hours of admission.

Previously reported cases have described tuberculous meningitis with hydrocephalus in immunocompetent adults and miliary tuberculosis with tuberculous meningitis, hydrocephalus, and severe hyponatraemia [15,16]. Compared with these reports, the present case adds a migrant-health and communication-safety dimension by showing how epidemiological risk, language discordance, metabolic abnormality, and evolving neurological signs can interact to delay recognition. It also has public health relevance because the patient had recently migrated, was not registered with a general practitioner, and lived with close contacts. Prompt public health notification and contact tracing are therefore essential in similar cases.

## Conclusions

This case highlights the catastrophic consequences of delayed recognition of tuberculous meningitis in an immunocompetent young adult. The combination of miliary tuberculosis, CNS involvement, hydrocephalus, and SIADH underscores the need for a high index of suspicion in migrants from endemic regions. Timely access to interpreter services, early diagnosis, anticipation of complications, and coordinated multidisciplinary care are essential to improving outcomes. Robust public health measures also remain crucial to preventing further transmission.
